# Single-cell analysis of a progressive Rosai–Dorfman disease affecting the cerebral parenchyma: a case report

**DOI:** 10.1186/s40478-024-01794-z

**Published:** 2024-05-20

**Authors:** Guo-Hao Huang, Guo-Long Liu, De-Zhi Huang, Xin-Wei Diao, Sheng-Qing Lv

**Affiliations:** 1 Department of Neurosurgery, Xinqiao Hospital, Army Medical University, Chongqing, 400037 China; 2 Medical Center of Hematology, Xinqiao Hospital, Army Medical University, Chongqing, 400037 China; 3 Department of Pathology, Xinqiao Hospital, Army Medical University, Chongqing, 400037 China

**Keywords:** Rosai–Dorfman disease, Central nervous system, Single-cell RNA sequencing, Histiocytes, KRAS mutation, Oligodendrocyte

## Abstract

Neurologic Rosai–Dorfman disease (RDD) is a rare type of non-Langerhans cell histiocytosis that affects the central nervous system. Most neurologic RDDs grow like meningiomas, have clear boundaries, and can be completely resected. However, a few RDDs are invasive and aggressive, and no effective treatment options are available because the molecular mechanisms involved remain unknown. Here, we report a case of deadly and glucocorticoid-resistant neurologic RDD and explore its possible pathogenic mechanisms via single-cell RNA sequencing. First, we identified two distinct but evolutionarily related histiocyte subpopulations (the C1Q+ and SPP1+ histiocytes) that accumulated in the biopsy sample. The expression of genes in the KRAS signaling pathway was upregulated, indicating gain-of-function of KRAS mutations. The C1Q+ and SPP1+ histiocytes were highly differentiated and arrested in the G1 phase, excluding the idea that RDD is a lympho-histio-proliferative disorder. Second, although C1Q+ histiocytes were the primary RDD cell type, SPP1+ histiocytes highly expressed several severe inflammation-related and invasive factors, such as WNT5A, IL-6, and MMP12, suggesting that SPP1+ histiocytes plays a central role in driving the progression of this disease. Third, oligodendrocytes were found to be the prominent cell type that initiates RDD via MIF and may resist glucocorticoid treatment via the MDK and PTN signaling pathways. In summary, in this case, we report a rare presentation of neurologic RDD and provided new insight into the pathogenic mechanisms of progressive neurologic RDD. This study will also offer evidence for developing precision therapies targeting this complex disease.

**Supplementary Information:**

The online version contains supplementary material available at 10.1186/s40478-024-01794-z.

## Introduction

Rosai–Dorfman disease (RDD) is a rare form of non-Langerhans cell histiocytosis (non-LCH) that is characterized by the accumulation of large pale CD68^+^, S100^+^, and CD1a^−^ histiocytes in various tissues and organs with a spectrum of clinical manifestations [1]. The incidence rate of RDD is approximately 1:200,000 [2]. Approximately 43% of patients present with extranodal involvement, and fewer than 5% had neurologic RDD; more than 90% had dura-based mass-mimicking meningiomas, and cerebral parenchyma involvement was sporadic [2]. Fewer than 300 cases of neurologic RDD have been reported in the literature worldwide [3]. RDD is considered a benign self-limiting disease, although a subset of patients may experience an aggressive course [1]. Treatment of neurologic RDD includes surgery (only for solitary disease), radiotherapy, steroids, and various targeted agents [1]. However, responses to steroids are variable and not durable, and little is known about the efficacy of targeted therapy for neurologic RDD due to the limited number of patients and the largely unknown molecular mechanisms underlying the development of this disease. Here, we present a rare case of neurological RDD with a progressive course and explore the possible pathogenic mechanisms by comprehensive single-cell RNA sequencing (scRNA-seq) analysis. Our findings will shed new light on these devastating RDDs and provide a basis for developing targeted therapies to improve patients’ lives.

## Case description

**Fig. 1:**
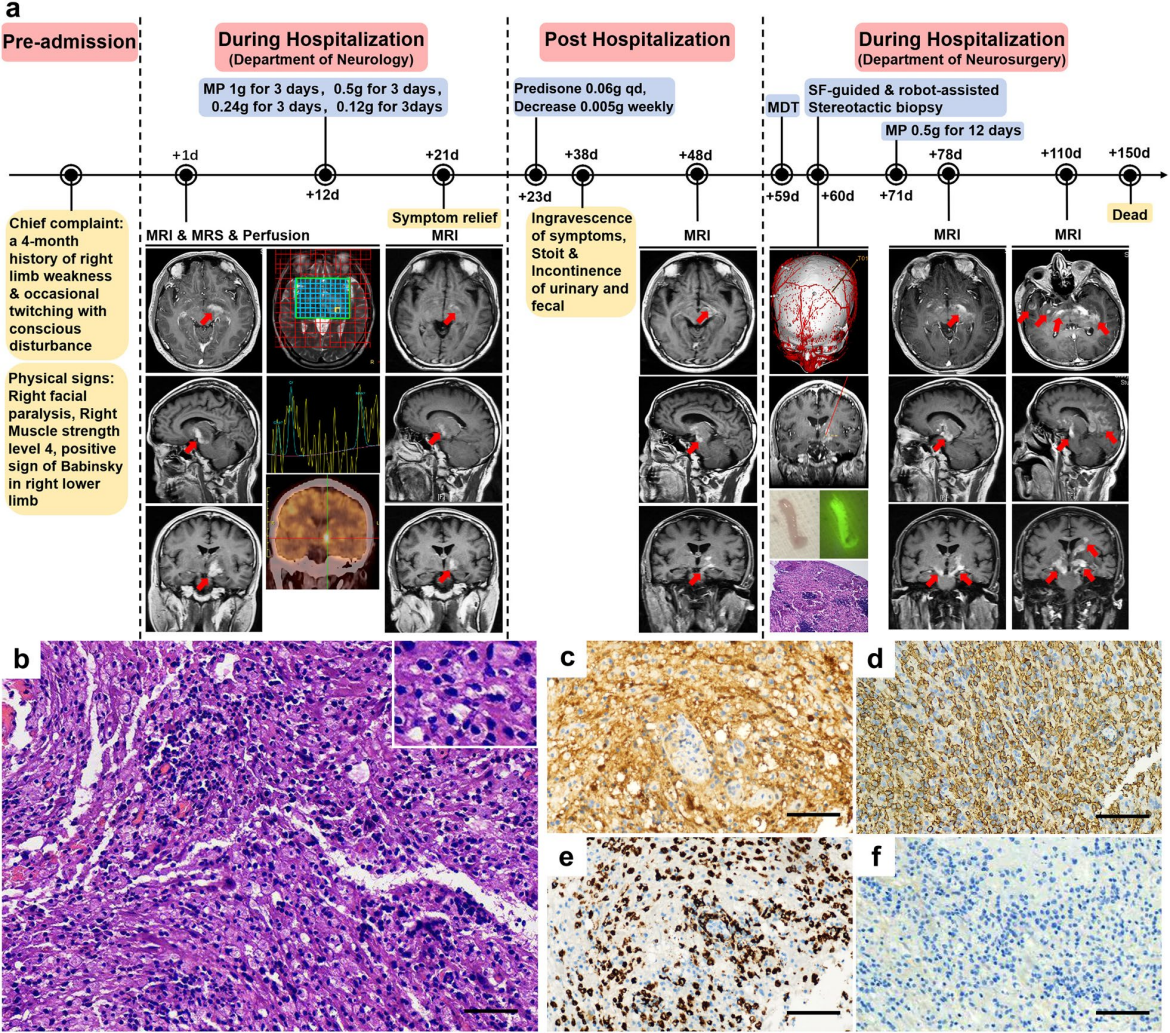
Patient’s clinical course and routine pathological stains. **a** Timeline of the clinical course for the patient with contrast-enhanced T1 MRIs demonstrating disease progression from the left basal ganglia, thalamus, and cerebral peduncle to multiple cerebral regions. **b** Hematoxylin–eosin (H&E) staining showing massive, large, and pale histiocytes that accumulated in the lesions. **c**–**f** Immunohistochemistry showing positive staining for S-100 (**c**), CD163 (**d**), and CD68 (**e**) and negative staining for CD1a (**f**) in the biopsy lesions. Red arrow, contrast-enhanced T1 lesions. MP, methylprednisolone. MDT, multidisciplinary team. SF-guided, sodium fluorescein-guided. Scale bars: 100 μm

A 54-year-old male patient presented with a 4-month history of right limb weakness and occasional limb twitching with unconsciousness (Fig. 1a). Electroencephalography confirmed the existence of epileptic waves. Magnetic resonance imaging (MRI) revealed equally enhanced signals on contrast-enhanced T1 in the left basal ganglia, thalamus, and cerebral peduncle. Both magnetic resonance spectroscopy (MRS) and positron emission computed tomography (PET-CT) were biased toward inflammatory disease (Fig. 1a). However, except for the positive serum IgG of Epstein‒Barr virus (EBV), antibodies for autoimmune encephalitis, demyelinating diseases, paraneoplastic syndrome, and oligoclonal bands from the patient’s serum and cerebrospinal fluid were negative. High-dose methylprednisolone (MP) was administered for diagnostic treatment (Fig. 1a). Afterwards, the patient’s clinical symptoms were partially relieved, and MRI showed that the range of the lesions decreased (Fig. 1a). The patient was then discharged and continued taking prednisone orally every day (Fig. 1a). Half a month later, the patient gradually started becoming unable to walk, and urinary and fecal incontinence developed. MRI revealed that the lesions increased in size and unilaterally extended to the brainstem and temporal lobe (Fig. 1a). The patient was admitted again and underwent sodium fluorescein-guided and robot-assisted stereotactic biopsy. Histopathologic analysis revealed the accumulation of many large and pale histiocytes with positive staining for S-100, CD163, and CD68 but negative staining for CD1a (Fig. 1b–f). A rare case of RDD extensively affecting the cerebral parenchyma was diagnosed by a senior pathologist. Although a high dose of MP was administered immediately after biopsy, the patient gradually deteriorated, and a repeat MRI showed that the lesions had bilaterally disseminated to multiple cerebral regions (Fig. 1a). Soon after, the patient lapsed into a coma, and his family members decided to stop treatment.

## ScRNA-seq and statistical analysis

**Fig. 2:**
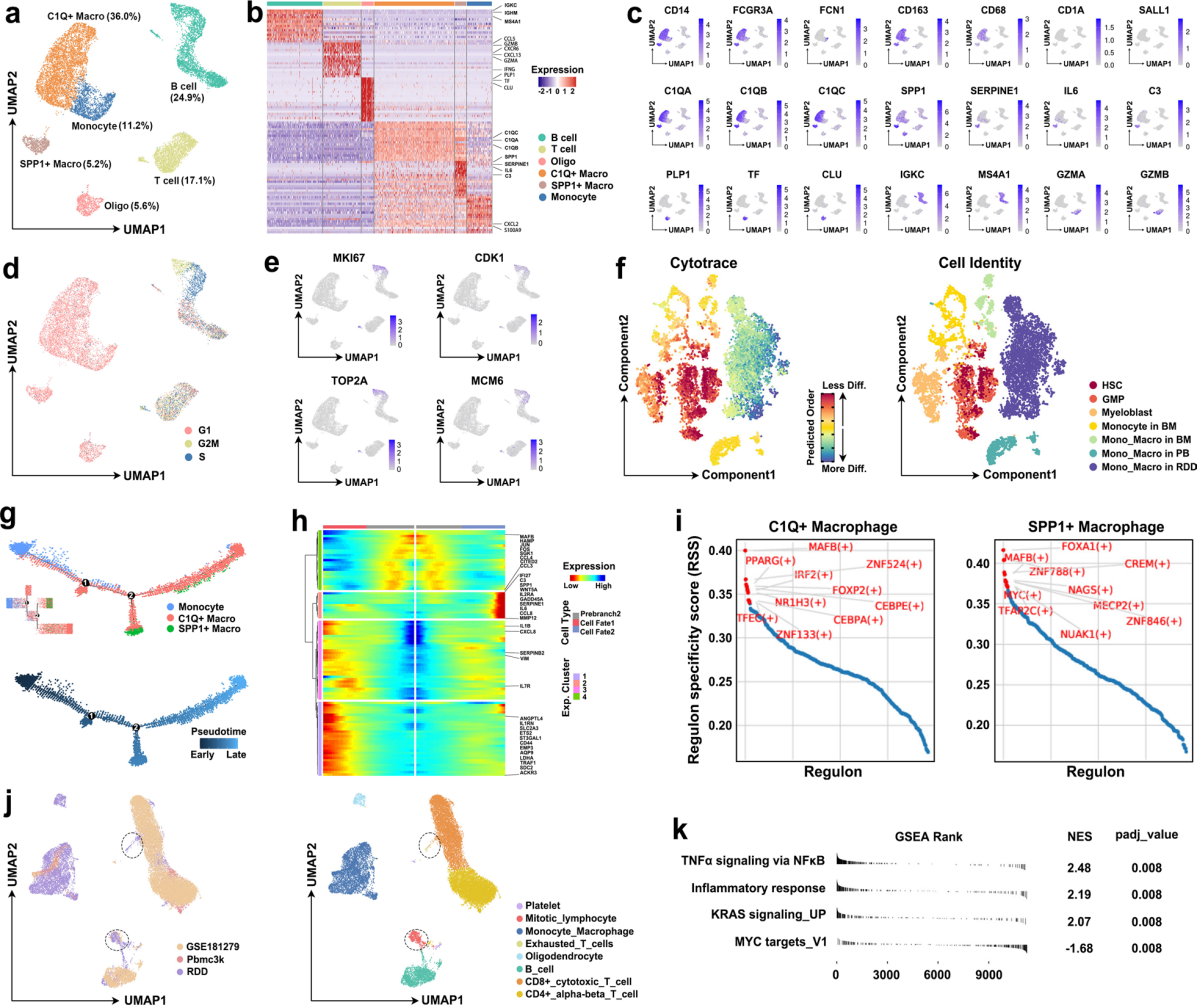
RDD is an inflammatory disease. **a** UMAP plot of the major cell types identified in the lesions, with color-coded cell clustering annotated based on marker gene expression. **b** Clustered heatmap showing the top differentially expressed genes across different cell types. **c** UMAP plot (from **a**) annotated with the expression of selected genes. **d** UMAP plot (from a) annotated with color-coded cells based on different phases of the cell cycle. **e** UMAP plot (from **a**) annotated with the expression of proliferation-related genes. **f** UMAP plot depicting the distribution of cytoTRACE scores (left) and cell identity (right) among different cell types. **g** Trajectory plot of monocytes, C1Q+ macrophages, and SPP1+ macrophages arranged according to color-coded cell type (upper) and pseudotime (lower). **h** Heatmap representing the smoothed expression of pseudotime‐dependent genes along pseudotimeeline at prebranch2. Pseudotime-dependent genes were clustered into 4 clusters according to different expression patterns. **i** Regulon-specific scatter plot of C1Q+ and SPP1+ macrophages. The top 10 regulons are highlighted in red. **j** UMAP plot annotated with cells from different datasets (left) and cell types (right). **k** GSEA showing the significantly enriched pathways in monocytes/macrophages from lesions compared with monocytes/macrophages from public normal datasets. Oligo, oligodendrocyte. Macro, macrophage. Diff., differentiated. HSCs, hematopoietic stem cells. GMP, granulocyte-monocyte progenitor. BM, bone marrow. PB, peripheral blood. Mono_Macro, monocyte and macrophage

To further investigate this progressive disease, we performed scRNA-seq of the biopsy, and 12,356 cells passed modularized quality control. By optimized clustering and annotation of these cells, we observed that there were no tumor cells but rather a large number of inflammatory cells, including macrophages (41.2%), monocytes (11.2%), B cells (24.9%), T cells (17.1%) and oligodendrocytes (5.6%), in the biopsied lesions (Fig. 2a–c). Markers of dendritic cells (CD1A and CD207) were almost negative in this lesion, excluding the diagnosis of LCH (Fig. 2c). Further clustering revealed two subpopulations of macrophages, one with high expression of C1QA, C1QB, and C1QC (C1Q+, 36.0%) and the other with high expression of SPP1 (SPP1+, 5.2%); both of these cell types are considered pathogenic histiocytes (Fig. 2a–c). As RDD is regarded as a lympho-histioproliferative disorder [2], we performed a *Cell Cycle* analysis to investigate this idea. The results showed that nearly all the histiocytes were in the G1 phase, and proliferation-related markers (MKI67, CDK1, TOP2A, and MCM6) were barely expressed (Fig. 2d–e), suggesting that these cells were more likely recruited from peripheral blood mononuclear cells (PBMCs) instead of tumoral cells undergoing sustained growth. Moreover, by utilizing the *cytoTRACE* algorithm to predict the developmental relationship between normal hematopoietic stem cells or their progenies (from the GEO dataset GSE120211) and histiocytes, we found that histiocytes were the most highly differentiated (Fig. 2f), confirming that these cells were recruited from PBMCs and endowed with a more specific function.

To explore the evolutionary relationship of the subpopulations of histiocytes, we applied unsupervised trajectory analysis using the *Monocle2* algorithm. Pseudotime analysis revealed a generally dynamic transition from monocytes to C1Q+ histiocytes and then to SPP1+ histiocytes (Fig. 2g). BEAM analysis revealed the gradual upregulated expression of a panel of genes related to sustained macrophage recruitment and migration, cytokine/chemokine production, phagocytosis, immunosuppression and degradation of the extracellular matrix (ECM) (SPP1 [4], C3 [5], WNT5A [6], IL6 [7], and MMP12 [8]) (Fig. 2h) during the emergence of SPP1+ macrophages, thus providing direct evidence of the inflammatory and progressive nature of this case. *PyScenic* analysis was then performed to investigate the upstream transcription factors (TFs) of the differentially expressed genes during the development of the histiocytes. The results showed that both C1Q+ and SPP1+ macrophages expressed mainly immunosuppressive-related TFs, such as MAFB [9] (Fig. 2i). Moreover, we integrated and compared public normal PBMC single-cell data from pbmc3k (from 10 × Genomics) and GSE181279 with the present data (Fig. 2j) and observed that there were more mitotic lymphocytes and exhausted T cells in these lesions than in healthy individuals (Fig. 2j), suggesting that while histiocytes can recruit lymphocytes and promote their proliferation, the normal functions of these lymphocytes were suppressed as well; this result is consistent with the immunosuppressive nature of C1Q+ and SPP1+ macrophages. Notably, GSEA of the differentially expressed genes between monocytes/macrophages from public health states and the present data showed that in addition to the upregulated expression of genes in the NF-κB and inflammatory response pathways in the lesions (Fig. 2k), which reflects the highly inflammatory state of this disease, genes in the KRAS signaling pathway also had significantly upregulated expression (Fig. 2k); these results indicated that the histiocytes, in this case, were likely harboring well-known gain-of-function mutations in KRAS, as previously described [1].

**Fig. 3:**
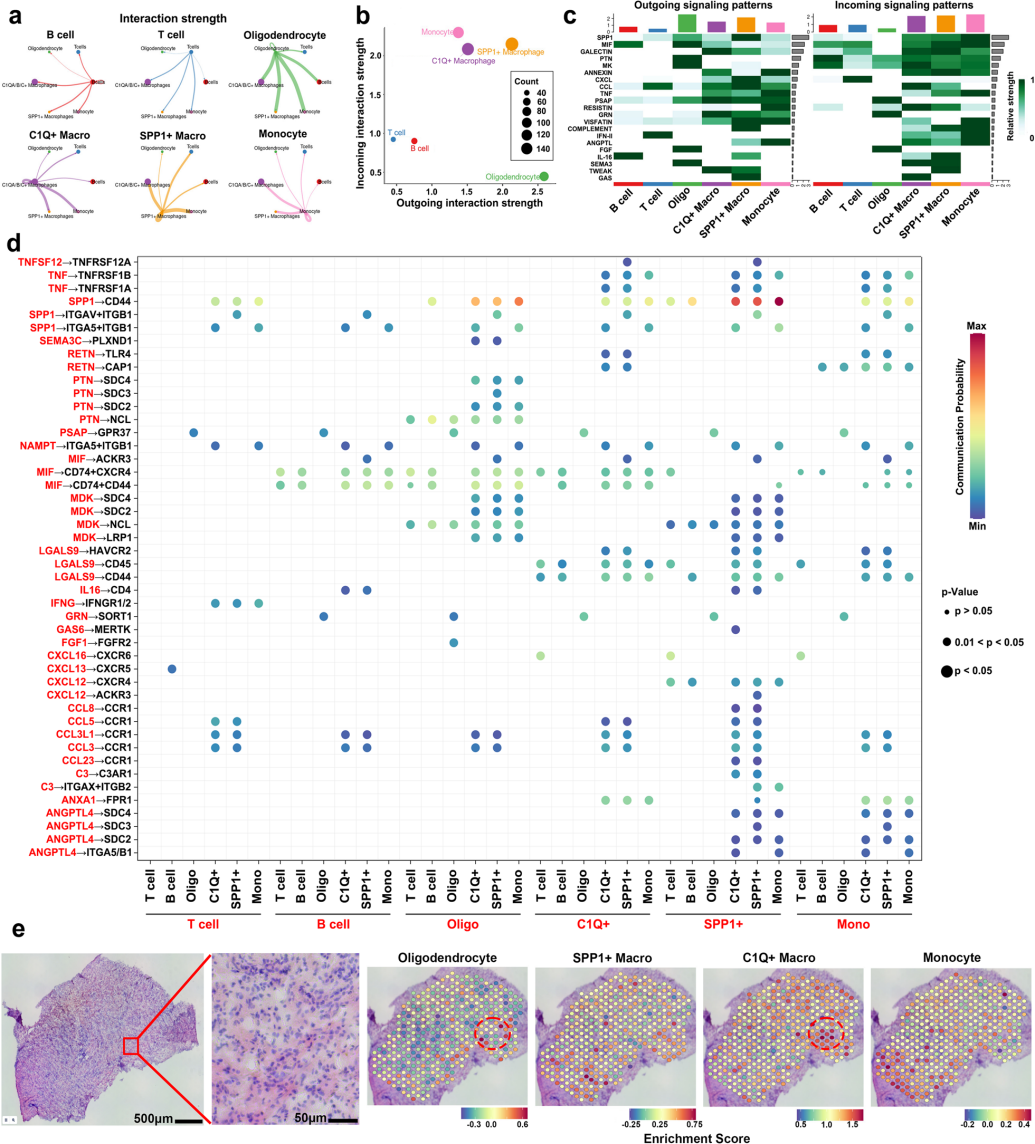
Oligodendrocytes and SPP1+ macrophages are the most prominent types of cells that mediate cell‒cell communication. **a** Circle plot showing the strengths of interactions between different cell types. **b** Scatter plot showing the cell types with different outgoing or incoming interaction strengths. The size of the dots represents the number of interactions. **c** Heatmaps of the overall outgoing (left) or incoming (outgoing) signaling patterns of each cell type in the biopsy lesion. **d** Dot plot of the probabilities of important ligand‒receptor pairs communicating between each cell type. The dot color reflects the communication probability, and the dot size represents the *p* value. **e** H&E staining of RDD samples for spatial transcriptomic (ST) sequencing and the distributions of oligodendrocyte, SPP1+ Macro, C1Q+ Macro, and monocyte signatures in the RDD ST dataset. Oligo, oligodendrocyte. Macro, macrophage. Mono, monocyte. C1Q+, C1Q+ macrophages. SPP1+, SPP1+ macrophages

Finally, *CellChat* analysis was performed to infer the interactions between the major cell types in these lesions. One of the most exciting discoveries is that oligodendrocytes contribute more substantially to outgoing interactions than other cell types (Fig. 3a–c), facilitate monocyte recruitment and the infiltration of other immune cells via the MIF signaling pathway [10], counteract glucocorticoid treatment, and activate the NF-κB pathway via the PTN and MK signaling pathways [11] (Fig. 3c–d), suggesting that oligodendrocytes may play vital roles in the initiation of this disease and the maintenance of glucocorticoid resistance. The other prominent cell type was SPP1+ macrophages, which possess both relatively strong outgoing and incoming interactions (Fig. 3a–b), indicating that these cells play a central role in the development and progression of this disease. Specifically, the most abundant signaling pathways in SPP1+ macrophages include SPP1, C3, and CXCL, which function through cytokine/chemokine production, phagocytosis, the M2 polarization of macrophages (SPP1 to CD44, especially to monocytes) [4], monocyte adhesion and activation (C3 to ITGAX + ITGB2) [5], and lymphocyte chemotaxis (CXCL12 to CXCR4) (Fig. 3c–d) [12]. Moreover, we found that monocytes promote robust incoming interaction strength, which is consistent with the above findings that showed that monocytes receive MIF, PTN, MDK, and SPP1 signals from other cell types to differentiate and participate in the initiation, maintenance, and progression of RDD (Fig. 3a–d). To further confirm the cell‒cell communication in situ, we performed spatial transcriptome analysis, and the results showed that oligodendrocytes and monocyte-macrophage lineages (especially C1Q+ macrophages) tended to colocalize (Fig. 3e), which is consistent with the robust interaction strength between oligodendrocytes and monocyte-macrophage lineages indicated by scRNA-seq analysis (Fig. 3a).

## Discussion and conclusions

Since its first description by Juan Rosai and Ronald Dorfman in 1969, RDD has been reported in more than 2000 papers; these reports mainly include the clinical and pathological features of RDD. The pathogenic mechanisms of RDD are still not fully understood, and several vital questions remain to be answered. For example, what are the characteristic differences between pathogenic histiocytes (especially for progressive RDD) and malignantly transformed cells? What are the mechanisms driving the accumulation of histiocytes in specific tissues? What are the interactions between histiocytes and other cellular components? What are their possible roles in the progression of RDD? The present report describes a rare case of disseminated and refractory neurologic RDD and its possible pathogenic mechanisms by conducting a comprehensive single-cell transcriptomic analysis, which allowed us to explore the diverse and discrete cellular populations in a complex disease sample. We think several fascinating discoveries that made in this report deserve further discussion.

First, RDD is considered a neoplastic process due to the discovery of recurrent somatic gain-of-function mutations in the oncogenic MAPK pathway (e.g., *NRAS*, *KRAS*, *MAP2K,* and *BRAF*) [1]. In the present case, although we did not perform whole-exon/genome sequencing (WES/WGS) because of the limited number of biopsy samples, we did find enrichment of the KRAS signaling pathway in the histiocytes, suggesting the presence of gain-of-function mutations in *KRAS*. Moreover, using the *Cell Cycle* and *CytoTrace* algorithm, we found that the histiocytes were highly differentiated and barely proliferated, suggesting that they were recruited from peripheral blood instead of being locally derived from the abnormal proliferation process of a neoplasm. This contradictory phenomenon may be explained by the proto-oncogene (e.g., *RAS, RAF*) mutation-related oncogene-induced senescence (OIS) and senescence-associated secretory phenotype (SASP), which can prevent cancer progression by halting the proliferation of oncogene-expressing cells and thus dampen malignant transformation [13]. At the same time, these phenotypes can also alert the immune system to eliminate oncogene-expressing cells, which, if persistent, may generate a chronic and detrimental inflammatory milieu [13].

Second, by transcriptomic and pseudotime analysis, we identified, for the first time, two distinct but evolutionarily related RDD subpopulations: C1Q+ and SPP1+ histiocytes. Moreover, we found that SPP1+ histiocytes were also C1QA/B/C positive and shared the same prominent TFs, such as MAFB, with C1Q+ histiocytes, demonstrating that the SPP1+ histiocytes were derived from C1Q+. Complement component 1q (C1Q), the initiating protein of the classical complement cascade, has been identified as a significant marker of a particular subpopulation of tissue-resident macrophages or tumor-associated macrophages (TAMs), which is often characterized by the coexpression of C1QA, C1QB, C1QC, HLA-DR, and APOE and is directly correlated with T-cell exhaustion and immune tolerance [14]. Secreted phosphoprotein 1 (SPP1), a major noncollagenous protein of bone, is overexpressed in a wide range of cancers and is correlated with poor prognosis [4]. In this report, we found that SPP1+ histiocytes highly express genes associated with severe inflammation, such as WNT5A [7]; immunosuppression and phagocytosis, such as IL6 [8]; and ECM degradation, such as MMP12 [9]. These genes may thus confer the central role of SPP1+ histiocytes in the progression of this disease.

Third, several studies have shown that oligodendrocytes are vulnerable to activating the transcription of immune response genes in specific disease contexts and play critical roles in initiating neuroinflammatory processes [15]. In this case, we found that oligodendrocytes include the strongest outgoing interactions and can interact mainly with the monocyte-macrophage lineage via the MIF-CD74, SPP1-CD44, PTN-SDC2/3/4, and MDK-NCL axes, promoting macrophage and other immune cell recruitment and glucocorticoid resistance, indicating that oligodendrocytes may play prominent roles in initiating and maintaining neurologic RDD. This finding based on the presence of oligodendrocytes in neuroinflammation will provide a significant basis for developing effective drugs that target the interaction between oligodendrocytes and histiocytes.

Overall, we hypothesize that this patient may have been born with innate or acquired *KRAS* mutations in monocytes before the initiation of this disease. When inflammation or injury accidentally occurs in the central nervous system, mutated monocytes are more susceptible to cytokines (such as MIF) that are secreted by resident cells (such as oligodendrocytes in this case) and are thus substantially recruited and differentiated to C1Q+ and then to SPP1+ macrophages, which can abnormally secrete SPP1, C3 and CXCLs to suppress normal lymphocyte function, degrade the ECM and extensively invade the brain parenchyma. We are optimistic that this hypothesis can be resolved by mechanistic experiments in animal models in the future.

The case reported here provides new insight into the development and progression of progressive neurologic RDD. These findings may provide evidence for developing precision therapies targeting *KRAS* mutations, SPP1+ macrophages, or interactions between resident cells and histiocytes.

### Supplementary Information

**Additional file 1.** This file includes a detailed description of the scRNA-seq protocol, analytic pipeline, settings, and references.

## Data Availability

The datasets used during the current study are available from the corresponding author on reasonable request.

## Abbreviations

RDD: Rosai–Dorfman disease
ScRNA-seq: Single-cell RNA sequencing
MRS: Magnetic resonance spectroscopy
PET-CT: Positron emission computed tomography
EBV: Epstein‒Barr virus
MP: Methylprednisolone
MDT: Multidisciplinary team
SF-guided: Sodium fluorescein-guided
PBMCs: Peripheral blood mononuclear cells
ECM: Extracellular matrix
TFs: Transcription factors
WES/WGS: Whole exon/genome sequencing
OIS: Oncogene-induced senescence
SASP: Senescence-associated secretory phenotype
TAMs: Tumor-associated macrophages
