# Supplementary material for: Single-cell analysis of a progressive Rosai–Dorfman disease affecting the cerebral parenchyma: a case report
Source: Acta Neuropathol Commun. 2024 May 20;12:78. doi: 10.1186/s40478-024-01794-z (PMC11103976; doi:10.1186/s40478-024-01794-z)
Supplement: Supplementary file 1 — Additional file 1. This file includes a detailed description of the scRNA-seq protocol, analytic pipeline, settings, and references. [file 40478_2024_1794_MOESM1_ESM.docx]

**Supplemental Methods:**

**Sample preparation and scRNA-seq.** Fresh lesions were harvested immediately from biopsy and stored in tissue preservation solution (Singleron Bio Com, Nanjing, China) for scRNA-seq. Cell isolation and library preparation for scRNA-seq were performed using the GEXSCOPE platform (Singleron Bio Com, Nanjing, China) as previously described [1]. Paired-end sequencing was performed based on the Illumina sequencing platform.

**Processing of scRNA-seq data.** The R software package “Seurat (version 4.3.0; <http://satijalab.org/seurat/>)” [2] was used to convert scRNA-seq sparse matrices into Seurat objects. Firstly, we filtered out the cells with < 300 UMIs detected, with either < 300 or > 5000 unique genes detected, with > 20% of UMIs from mitochondrial genes, or with > 1% of UMIs from hemoglobin genes. After that, we retained 12356 cells for further analysis. Secondly, scRNA-seq data was normalized using the “LogNormalize” method, and the top 20 principal components (PCs) were extracted based on the top 2000 highly variable genes. Thirdly, we performed Louvain clustering based on the top PCs using “FindNeighbors” and “FindClusters”, with the resolution parameter set to 0.5. Then, we performed Uniform Manifold Approximation and Projection embedding (UMAP) using the “RunUMAP” function for two-dimensional reduction and visualization. Lastly, we annotated cells based on the known cell type markers and differentially expressed genes between clusters identified by “FindAllMarkers”.

**Cell Cycle Analysis.** The cell cycle-related gene lists of S-phase and G2M-phase were obtained from “Seurat (version 4.3.0)” and matched with the filtered genes in the scRNA-seq data of the lesion. Then, the cell cycle scores of S-phase, G2M-phase, and G1-phase for every cell of the lesion were obtained by using “CellCycleScoring” and were visualized using “Dimplot”.

**Gene Set Enrichment Analysis (GSEA).** To uncover the aberrantly expressed signaling pathways of the monocytes/macrophages in the lesion, we downloaded normal peripheral blood mononuclear single-cell datasets from the Gene Expression Omnibus dataset (GSE181279) and the 10x genomics company (pbmc3k). The batch effects were removed by the R package “Harmony (version 0.1.1)”. After the conventional Seurat data processing pipeline, cells annotated as monocytes/macrophages were extracted, and genes differentially expressed between monocytes/macrophages from different datasets were identified using “FindMarkers”. Lastly, GSEA analysis was performed using the “fgsea” R package (species = “Homo sapiens”, category = “H”)^[3]^.

**CytoTRACE Analysis.** To infer the developmental hierarchy relationship between normal hematopoietic stem cells and their progenies and the monocytes/macrophages from the lesion, we downloaded the normal hematopoietic stem cells and their progenies single-cell datasets from the GSE120211 and further integrated it with the abovementioned integrated data for GSEA analysis by “Harmony (version 0.1.1)”. Then, the matrix of raw UMI counts and the annotation metadata from the integrated data was extracted and used as the input and analyzed by the “CytoTRACE (version 0.3.3)’ following the default parameters^[4]^.

**Trajectory analysis.** The pseudotime trajectory analysis was performed using the R package “Monocle2 (version 2.30.0)”^[5]^. The Seurat object containing the 'Monocyte', 'C1QA/B/C+ Macrophages', and 'SPP1+ Macrophages' cell types were generated with the “Subset” command from the “Seurat” R package. The matrix data was derived from the raw UMI counts of the processed Seurat object and used as the inputs. The newCellDataSet function of “Monocle2” was applied to create an object with the default parameters. Only genes with a mean expression ≥ 0.1 were used for dimension reduction by using the “reduceDimension” function with the following parameters: method = “DDRTree”, max_components = 2. The cells were then ordered and visualized with the “plot_cell_trajectory” function. To identify the gene panels associated with the differentiation of monocytes along different branches, the “Branch Expression Analysis Modeling (BEAM)” analysis was performed using the “BEAM” function, and genes differentially expressed at branch_point 2 with a q-value < 1e-4 were separated into 4 clusters and visualized with the “plot_genes_branched_heatmap” function.

**Single-cell regulatory network inference and clustering (SCENIC).** To identify cell type-specific regulatory networks, pySCENIC (version 0.10.2) was performed^[6]^. Briefly, genes in RcisTarget’s human feather databases were utilized to determine the search space around the transcription starting sites (TSS). These genes are predicted in a region 10 kb or 500 bp upstream and 10kb or 100 bp downstream of the TSS using the hg38 human reference genome. Then, we performed pySCENIC analysis with default parameters.

**Cell-Cell Communication Analysis.** The cell-cell communication analysis was performed using the R package “CellChat (version 1.6.1)”^[7]^. “Secreted Signaling” from CellChatDB was selected for analysis. The interactions between cells were identified and quantified based on the differentially over-expressed ligands and receptors for each cell group using “identifyOverExpressedGenes”, “identifyOverExpressedInteractions” and “computeCommunProb” functions. Differences in the strength of intercellular communications were calculated with the function “netVisual_circle”. The primary senders and receivers for cell-cell communication were identified with the “netAnalysis_signalingRole_scatter”. The top aggregated outgoing and incoming signaling pathways of major cell types were inferred by the “netAnalysis_signalingRole_heatmap” function. Specific ligand-receptor pairs contributing the most to the outgoing or incoming signaling pathways were analyzed with the “netAnalysis_contribution” function and visualized with the “netVisual_bubble” function.

**Processing of Spatial Transcriptomic sequencing data.** The H&E staining image was aligned using the manufacturer-provided st-pipeline (github.com/SpatialTranscriptomicsResearch/st_pipeline). Interested areas and matching position spots’ barcodes were selected using the Loupe Browser 7.0.1. Seurat v4.3.0 was used to sample the selected spots and normalize gene expression values using the variance stabilizing transformation method implemented in the “SCTransform” function. Differential expression between the major cell types in scRNA-seq was performed using the FindAllMarkers function in Seurat with default parameters.Spatial cell type signature scoring was performed using the AddModuleScore function in Seurat with default parameters based on the top 100 differentially expressed genes (adjusted p-value < 0.05 by Wilcoxon Rank Sum test).

**References:**

[1] Y. Zhou, D. Yang, Q. Yang, X. Lv, W. Huang, Z. Zhou, et al, Single-cell RNA landscape of intratumoral heterogeneity and immunosuppressive microenvironment in advanced osteosarcoma, Nat Commun 11(1) (2020) 6322. doi: 10.1038/s41467-020-20059-6.

[2] A. Butler, P. Hoffman, P. Smibert, E. Papalexi, R. Satija, Integrating single-cell transcriptomic data across different conditions, technologies, and species, Nat Biotechnol 36(5) (2018) 411-420. doi: 10.1038/nbt.4096.

[3] A. Liberzon, A. Subramanian, R. Pinchback, H. Thorvaldsdottir, P. Tamayo, J.P. Mesirov, Molecular signatures database (MSigDB) 3.0, Bioinformatics 27(12) (2011) 1739-40. doi: 10.1093/bioinformatics/btr260.

[4] G.S. Gulati, S.S. Sikandar, D.J. Wesche, A. Manjunath, A. Bharadwaj, M.J. Berger, et al, Single-cell transcriptional diversity is a hallmark of developmental potential, Science 367(6476) (2020) 405-411. doi: 10.1126/science.aax0249.

[5] C. Trapnell, D. Cacchiarelli, J. Grimsby, P. Pokharel, S. Li, M. Morse, et al, The dynamics and regulators of cell fate decisions are revealed by pseudotemporal ordering of single cells, Nat Biotechnol 32(4) (2014) 381-386. doi: 10.1038/nbt.2859.

[6] B. Van de Sande, C. Flerin, K. Davie, M. De Waegeneer, G. Hulselmans, S. Aibar, et al, A scalable SCENIC workflow for single-cell gene regulatory network analysis, Nat Protoc 15(7) (2020) 2247-2276. doi: 10.1038/s41596-020-0336-2.

[7] S. Jin, C.F. Guerrero-Juarez, L. Zhang, I. Chang, R. Ramos, C.H. Kuan, et al, Inference and analysis of cell-cell communication using CellChat, Nat Commun 12(1) (2021) 1088. doi: 10.1038/s41467-021-21246-9.
